# Using the near field optical trapping effect of a dielectric metasurface to improve SERS enhancement for virus detection

**DOI:** 10.1038/s41598-021-85965-1

**Published:** 2021-03-25

**Authors:** Cameron F. Kenworthy, L. Pjotr Stoevelaar, Andrew J. Alexander, Giampiero Gerini

**Affiliations:** 1 Optics Department, The Netherlands Organization for Applied Scientific Research, TNO, 2628CK Delft, The Netherlands; 2 School of Chemistry, The University of Edinburgh, Edinburgh, EH9 3JJ UK; 3 TU/e, Electromagnetics Group, Eindhoven University of Technology, 5600MB Eindhoven, The Netherlands

**Keywords:** Nanophotonics and plasmonics, Metamaterials, Optical manipulation and tweezers, Raman spectroscopy

## Abstract

In this paper, we report the effect of optical trapping on the enhancement factor for Raman spectroscopy, using a dielectric metasurface. It was found that a higher enhancement factor (up to 275%) can be obtained in a substrate immersed in water, where particles are freee to move, compared to a dried substrate, where the particles (radius $r=9$ nm, refractive index $n=1.58$) are fixed on the surface. The highest enhancement is obtained at low concentrations because, this case, the particles are trapped preferentially in the regions of highest electric field (hotspots). For high concentrations, it was observed that the hotspots become saturated with particles and that additional particles are forced to occupy regions of lower field. The dielectric metasurface offers low optical absorption compared to conventional gold substrates. This aspect can be important for temperature-sensitive applications. The method shows potential for applications in crystal nucleation, where high solute supersaturation can be achieved near the high-field regions of the metasurface. The high sensitivity for SERS (surface-enhanced Raman spectroscopy) at low analyte concentrations makes the proposed method highly promising for detection of small biological particles, such as proteins or viruses.

## Introduction

Raman spectroscopy is an effective technique for reliable label-free molecular detection and identification. This is useful for a wide range of applications including medical diagnostics, forensics and defense. The ability to detect and identify molecules from small samples can be used to quickly and accurately pinpoint the signatures of infectious agents, which could be valuable for hard-to-detect species, such as viruses. Other sensing applications include identifying pigments, trace elements of explosives or narcotics, and toxic agents or environmental pollutants.

In traditional Raman spectroscopy, incident radiation is inelastically scattered, and provides the vibrational spectrum of an analyte; however, this is a very weak phenomenon with only 1 in $10^6-10^{10}$ photons exciting vibrational modes^1^. Surface-enhanced Raman spectroscopy (SERS) is used to boost the scattered signal power, by increasing the electric-field intensity local to the analyte^2^. SERS can be achieved using a nano-structured substrate, such as a metasurface, to produce regions of high electric field (hotspots)^3^. For analyte molecules in these field hotspots, the SERS signal can be increased by an enhancement factor of up to $10^8$^ 4^. The hotspots are, however, highly localized to specific regions of the substrate; only a small proportion of the analyte experiences the high field, leading to $\sim 2\%$ of the analyte producing $\sim 98\%$ of the SERS signal^5^.

In the majority of cases, metasurface substrates are plasmonic in nature, making use of localized surface-plasmon resonances (LSPRs) to provide the field enhancement near the surface. These oscillations in electron density can be induced by incident radiation coupling with the free electrons in a metallic substrate^6^. The incident radiation is absorbed by free electrons close to the metal surface, providing the additional kinetic energy for the LSPRs. This can lead to not only loss of signal power, but Joule heating of the metal and its local environment, which has the potential to perturb or even damage both the substrate and analyte^7^.

As proposed by Caldarola et al., dielectric metasurfaces have the potential to produce SERS enhancement equivalent to that of plasmonic substrates^7^. By comparison to conventional noble-metal nanostructures, one clear advantage of the dimer metasurface is the reduced heating due to optical absorption. The concept of a dielectric metasurface was developed further, by Černigoj et al., who designed an array of silicon nanopillar dimers (i.e., pairs of pillars) above a gold mirror, which was shown to provide a large SERS enhancement factor when the dimensions were optimized^8^. The coupling of Mie resonances induced between two cylindrical nanopillars produces a region of increased field intensity in the gap between the pillars.

This dimer field enhancement (DFE) resonance provides a field enhancement for a range of incident frequencies, and is tuneable by adjusting the dimer dimensions^8^. This allows the enhancement to be tuned to the range of emission frequencies of the Raman scattered radiation. Coupling also occurs between the dimers in the array, producing a sharp, narrowband, array field enhancement (AFE). This can be adjusted by changing the period of the array, and can be tuned to the wavelength of the incident light source for maximum enhancement^8^. The AFE and DFE being largely uncoupled geometrically allows for the resonances to be tuned independently to provide maximum enhancement for a particular analyte species.

The SERS enhancement factor (EF) calculated by Černigoj et al. assumes a uniform distribution of analyte particles immobilized over a dry surface (e.g., in air); therefore only a small percentage of particles by chance fall within the enhanced field regions. If a larger number of the analyte particles were present in the highest field regions, the EF would be greatly increased. The aim of the present work is to investigate optical trapping to localize analyte particles in high-field regions of nanopillar dimers in a metasurface array.

Nanoantenna structures have been used in fluid to produce the electric field gradients necessary for dielectrophoresis of nanoscale particles^9^. For particles with refractive index greater than the surrounding fluid, the field gradient provides a force in the direction of increasing field intensity, driving the particles to the highest field regions. Performing the SERS measurement in fluid allows the analyte particles to explore the liquid volume above the metasurface. This is unlike taking the measurement in air, where the particles would only be fixed on the substrate surface: therefore, a modification to the computation of the EF is required. The modified expression for calculating EF in a volume is given in the Methods section.

As we shall discuss below, a novel application of field-enhanced trapping is to induce crystal nucleation, as demonstrated by Masuhara, Sugiyama and co-workers^10–12^. A significant application where our proposed SERS enhancement method could be particularly important, is in the detection and identification of biological macromolecules, proteins, viruses, or virus fragments^13,14^.

## Results

**Figure 1:**
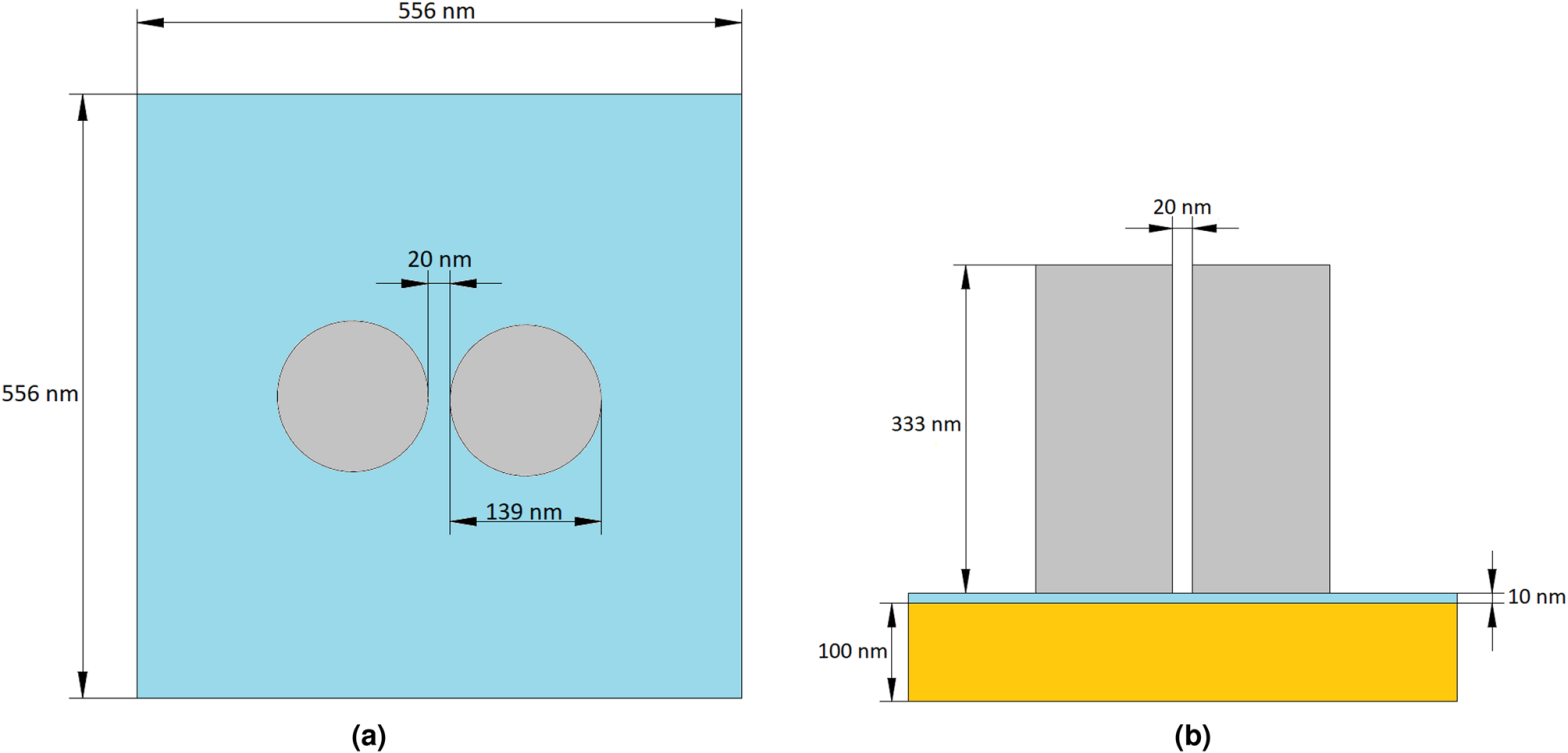
(**a**) Top view of a unit cell of the infinite array employed in numerical simulations. The unit cell (556 × 556 nm) incorporates a dimer consisting of two crystalline silicon pillars separated by a 20 nm gap. The two pillars are symmetrically displaced with respect to the center of the unit cell. (**b**) Side view of a unit cell. The substrate consists of a 10 nm thick fused quartz spacer above a 100 nm thick gold mirror. The array is immersed in water^15^.

In this section, we present a dielectric nanopillar dimer array for optimized performance in water. For details of the simulation and optimization procedures, see the Methods section. The substrate consists of a 100 nm thick gold mirror, coated with 10 nm of fused quartz (SiO$_2$), above which the array of silicon dimers is located. The low refractive index dielectric layer is used to reduce the excitation of surface plasmon polaritons at the interface with the gold layer due to scattering of the dimers. A square unit cell of 556 × 556 nm is used, with two silicon nanopillars spaced by 20 nm, as shown in Fig. 1. The pillars height is 333 nm and the diameter 139 nm.

**Figure 2:**
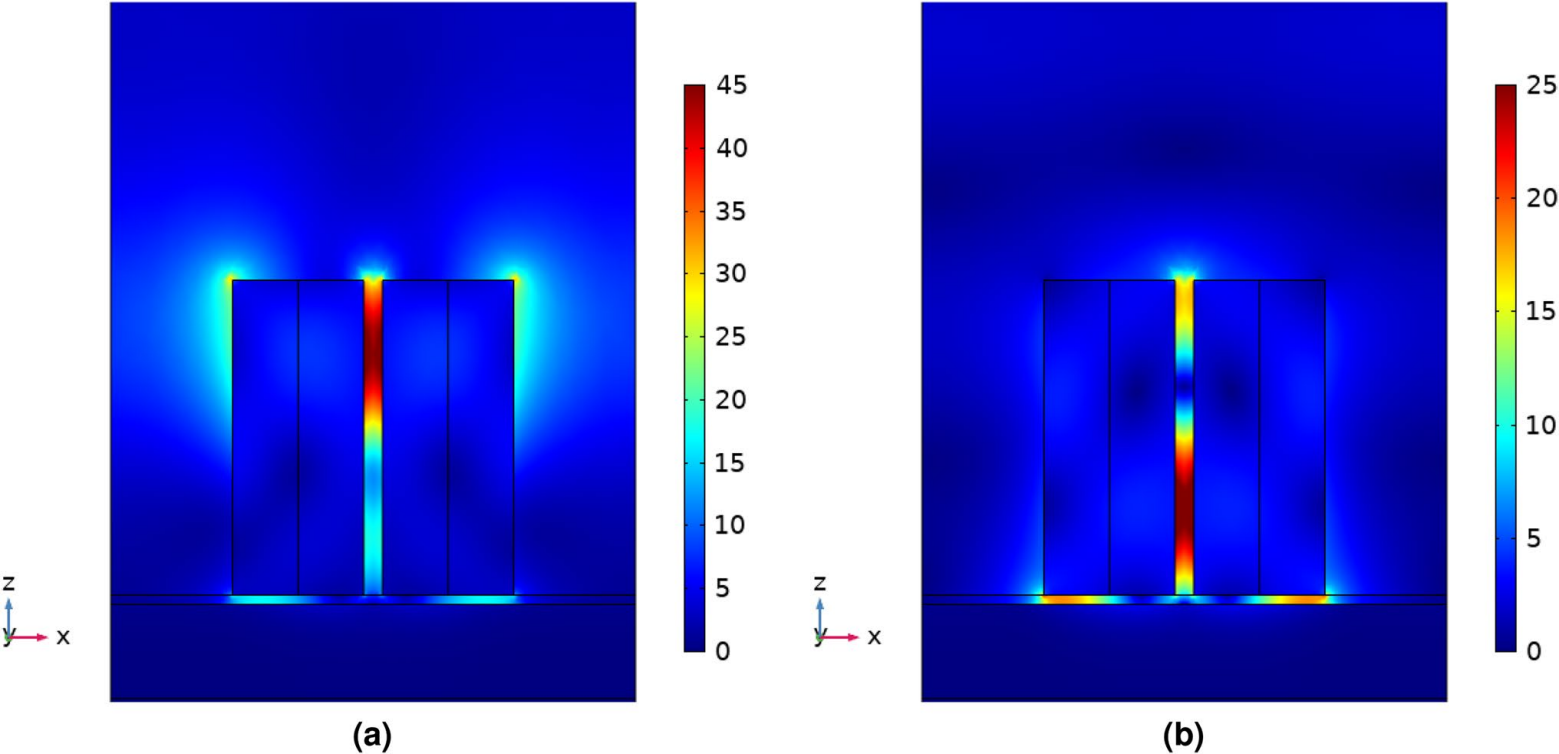
Scattered field magnitude when a plane wave is incident from above polarized parallel to the axis through the centre of both cylindrical nanoparticles with wavelength (**a**) 785 nm, the SERS excitation wavelength and (**b**) 850 nm, the center of the SERS emission band^16^. The spatial locations of enhanced field are almost identical to the case in air^8^, but we find that the magnitudes of enhancement are reduced, as shown in Fig. 3.

**Figure 3:**
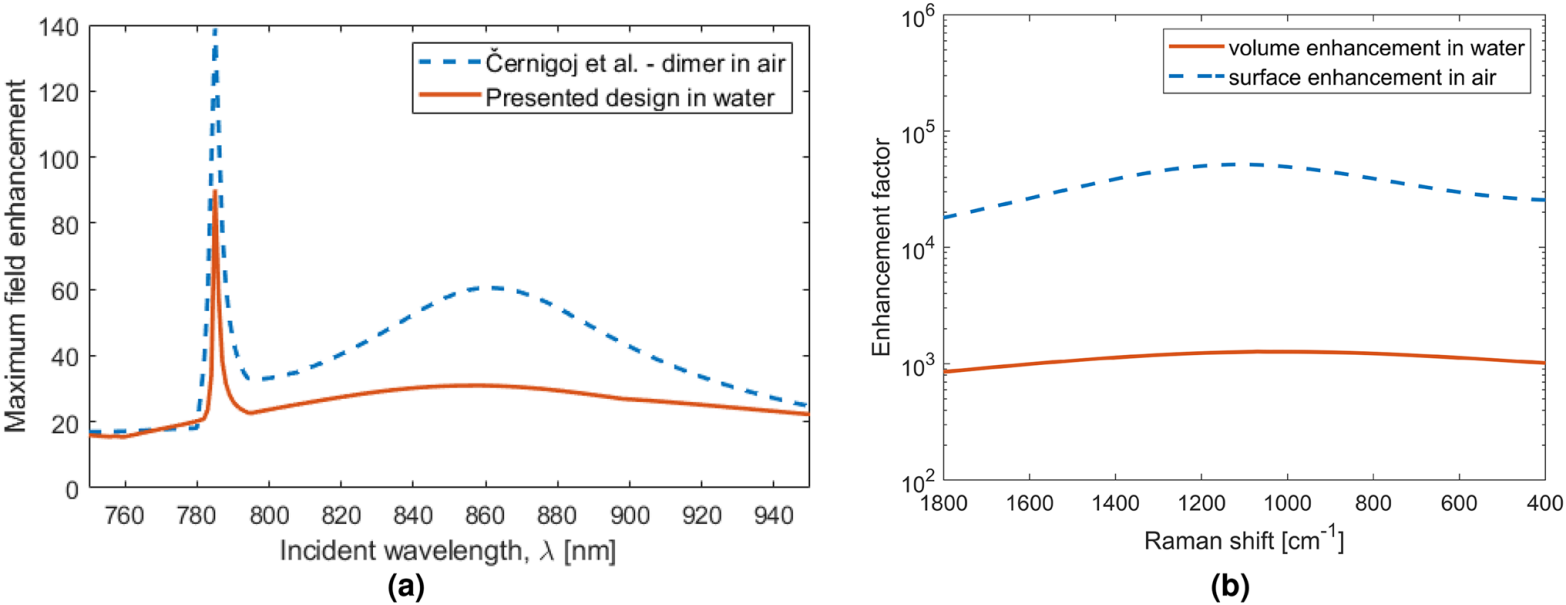
(**a**) Maximum local field enhancement within the unit cell of the crystalline silicon dimer metasurface in water and air, with plane wave incident light polarized along the axis through the centre of both cylindrical nanoparticles. Sweeping incident wavelengths from 750 to 950 nm, shows a narrow peak at 785 nm due to the lattice resonance, the array field enhancement (AFE) and a broad peak at 862 nm due to the dimer resonance, the dimer field enhancement (DFE). (**b**) Enhancement factor using a excitation wavelength of 785 nm, averaged over the unit cell fluid volume for the SERS substrate in water and averaged over the surface of the SERS substrate in air, for Raman shift values between 400 and 1800 cm$^{-1}$.

The scattered electric field distribution produced by the metasurface is shown in Fig. 2. The metasurface was illuminated with a normally incident plane wave polarized along the *x*-axis, as indicated in the figure. The configuration shown in Fig. 2 provides the required sharp electric field resonance at the SERS excitation wavelength (785 nm), and broad resonance around 850 nm in the SERS emission wavelength region. The maximum field enhancement occurs in the hotspot between the nanodimers: the results for the array immersed in water is shown in Fig. 3a, compared to the maximum field enhancement by the metasurface of Černigoj et al. in air.

The results presented in Fig. 3a show that the maximum field enhancement possible in air is more than 50% larger, for the majority of the wavelength range of interest, than what is achievable in water. The difference between the refractive indices of crystalline silicon and air is larger than the difference between the refractive indices of silicon and water. Therefore, stronger resonances between dimers are achieved in air. This is, however, not the only factor that contributes to the magnitude of the EF. Assuming a uniform distribution of analyte particles within the unit volume, the EF was calculated and is presented as a function of Raman shift in Fig. 3b, in comparison to the EF for molecules immobilized at the air-array interface achieved by Černigoj et al.^8^.

When analyte particles are suspended in fluid, they can move freely within the volume. Taking into account the gradient force, the distribution of particles is no longer uniform, with a higher density of particles in the high-field regions than the low-field regions. To model this distribution, an algorithm was developed to track the position of particles under the influence of the electric field distribution. Details of this modeling tool, including relevant equations, are given in Methods. Taking into account random thermal noise (i.e., Brownian motion), gravity, and Stokes drag, the modeling tool can predict the effect of the localized field enhancement produced by the dimer metasurface. The resulting distribution can be used to calculate the effect of the optical confinement on the EF of the SERS substrate. By releasing a regular array of particles within the unit cell and tracking their position over time, under the influence of the external forces, the individual EF for each particle can be calculated, in its initial position and at the position after a certain amount of time has passed. By using a large number, and summing over all particles, the total volumetric EF of the system can be estimated using Eq. 12 given in the Methods.

**Figure 4:**
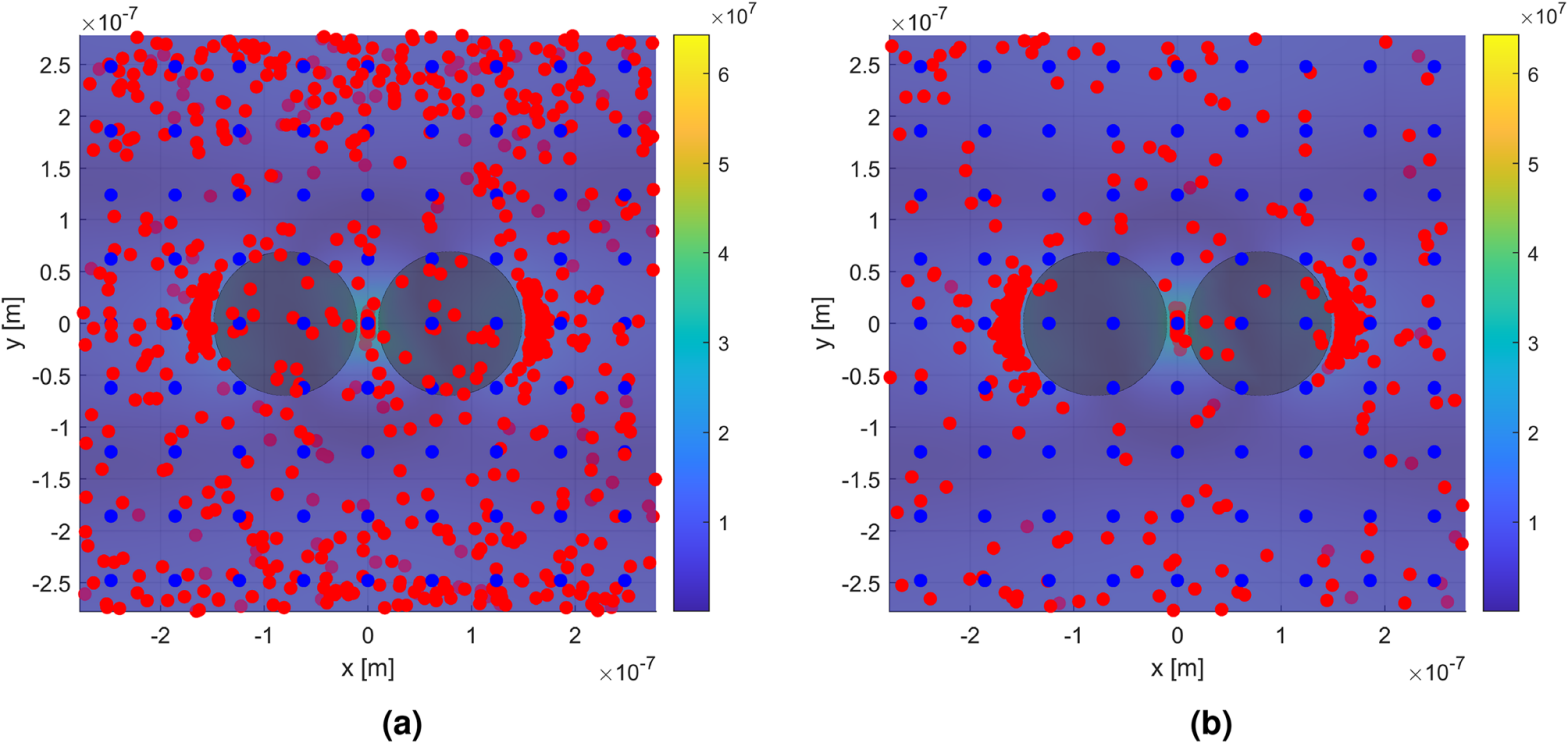
Top view of a single unit cell illustrating the trapping effect of the local field enhancement. The color scale indicates the electric field magnitude. The blue dots represent the initial particle positions in a regular array. The red dots represent the particle positions after (**a**) 1 ms, and (**b**) 10 ms, under influence of the external field. Dots with a slightly darker color indicates that they are behind the plane where the field magnitude is shown. The external field was produced by a plane wave incident from above with wavelength 785 nm and power 50 mW μm$^{-2}$, polarized along the axis through the centers of both nanoantennas (*x*-axis). The number of particles in initial positions appears lower due to their vertical alignment (along *z*) in the array^19^. The distributions of particles distance from the hotspot are shown in the Supporting Information.

For example, picornaviruses are non-enveloped RNA viruses, with radius approximately 11-15 nm that include enteroviruses such as polio and rhinovirus^17^. The size and refractive index of the poliovirus ($r \approx 15$ nm, $n = 1.61\pm 0.06$)^18^ are close to the size and refractive index of polystyrene particles ($r = 9$ nm, $n = 1.58$) employed in the present simulations. Therefore, 972 polystyrene nanospheres were released within the unit cell, in a regular 9 × 9 × 12 array, and the positions tracked over time. Figure 4 shows the initial positions (blue dots), and the positions after 1 and 10 ms (red dots), respectively, under the influence of a 50 mW μm$^{-2}$ incident plane wave.

**Figure 5:**
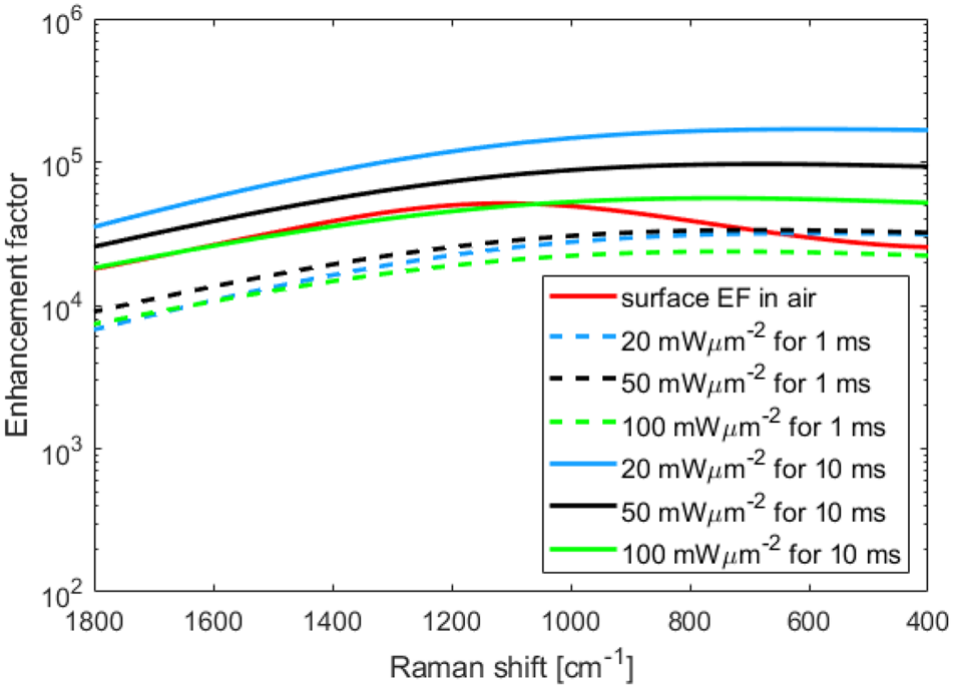
Calculated enhancement factors after 1 ms (dashed lines) and 10 ms (solid lines) of an incident plane wave (wavelength 785 nm) with intensity 20, 50 and 100  mW μm$^{-2}$, respectively. The integrated surface enhancement factor in air (*cf.* Fig. 3b) (solid red line) is shown for comparison.

Figure 5 shows the calculated initial and final EF (after 1 ms and 10 ms) with incident plane-wave powers of 20, 50, and 100 mWμm$^{-2}$, respectively. The integrated surface EF in air (*cf.* Fig. 3b) is shown for comparison.

**Figure 6:**
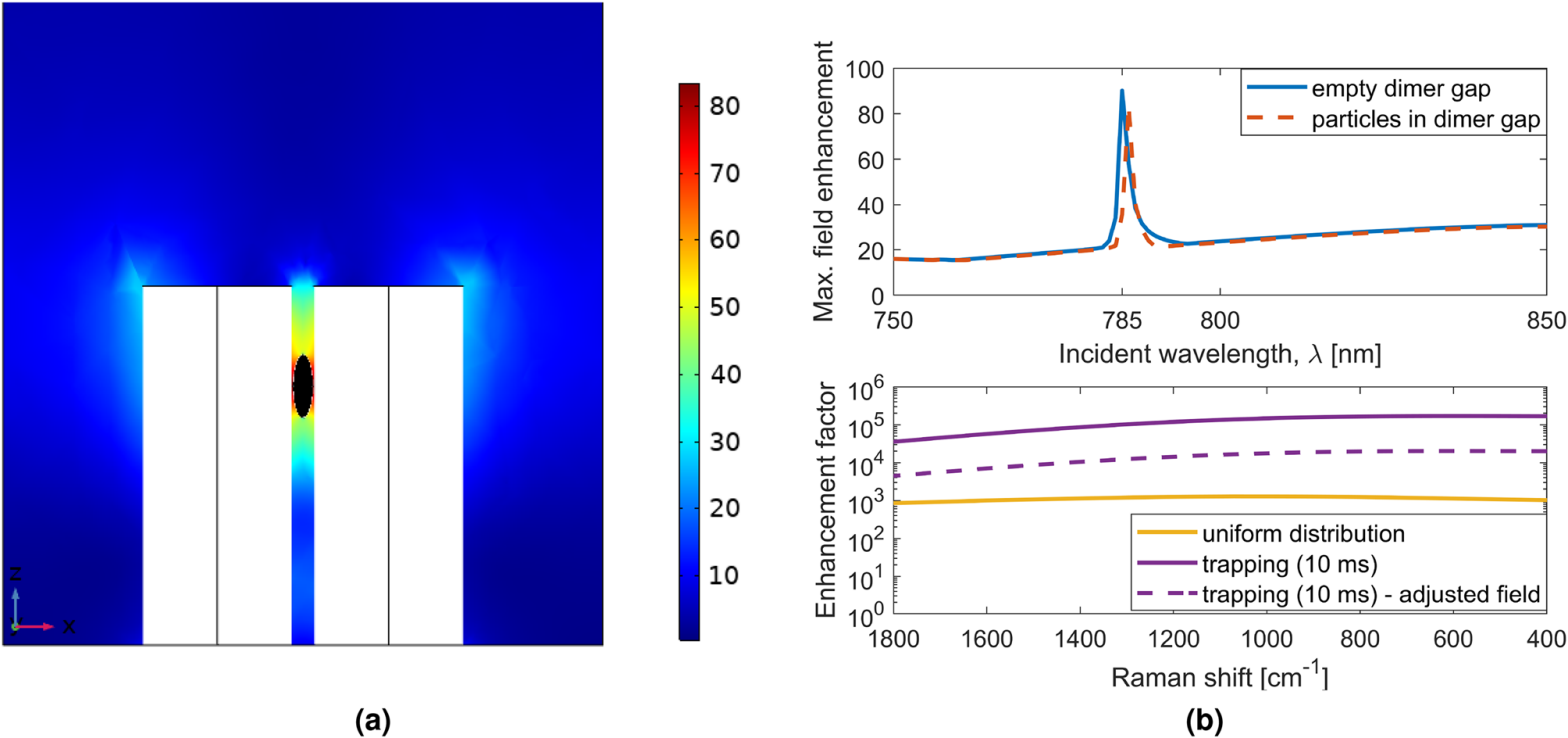
(**a**) Scattered electric field magnitude (incident wavelength 785 nm) showing a polystyrene ellipsoid (black) at the point of highest field magnitude^16^. The ellipsoid was chosen to simulate the presence of a few particles trapped within the dimer. In (**b**) the effect of including the polystyrene ellipsoid is shown: (top) the resulting maximum local field enhancement within the unit cell of the metasurface; (bottom) the SERS enhancement factor  (source: 20 mW μm^-2^, 785 nm).

The model used assumes a static electric field and does not take into account the effect of the particles on the field over time. One potential issue with this model is that, if the gap between the dimers is filled with a large number of particles, the field enhancement could be reduced. This effect was investigated by placing a polystyrene ellipsoid, with a volume equivalent to that of approximately 6 particles, at the highest field region for the laser excitation wavelength (785 nm), as shown in Fig. 6. Since the optical trapping is induced by the laser source, the particles are expected to localise around the hotspot at the laser wavelength. The resulting maximum field enhancement is shown in Fig. 6b (top). A small shift of +0.7 nm in the lattice resonance (AFE) peak wavelength is observed. Away from this resonance (785 nm), the position of the high field spots is different in the structure, as illustrated in Fig. 2b. Therefore, presence of particles near top of the dimer due to the laser source, do not impact the field distribution of maximum field enhancement. The shift in resonance leads to the EF being almost an order of magnitude lower when a 20 mW μm$^{-2}$, 785 nm source is used, as shown in Fig. 6b (bottom).

## Discussion

### Particle overlap

The tracking algorithm we have used tracks the centers of particles, and therefore allows for particles to overlap in space. For infinitely hard particles, this would of course produce nonphysical results. However, the method allows us to examine cases where particles can be considered soft, e.g., biological macromolecules; or are able to aggregate or merge, e.g., nanodroplets of solute as precursors for crystal nucleation^20,21^. To quantify the overlap, the percentage of particles that were separated by less than one diameter from the nearest neighbor was calculated. By taking random draws of a fixed number of particles from the spatial distribution after trapping, the percentage overlap for different concentrations was estimated, as shown in Table 1.

For a trapping period of 10 ms the overlap percentages are above 37%, indicating that particles will tend to aggregate. For a trapping time of 1 ms, the percentage overlap is noticeably lower for equivalent concentration and input power. This difference in overlap is consistent with the observation that laser-trapping crystallization requires longer trapping times^10,12^. For a trapping time of 1 ms it can be seen (Table 1) that the percentage overlap is strongly dependent on concentration. For an input power of 20 mW μm$^{-2}$ the percentage overlap doubles as the concentration increases by an order of magnitude.

For hard spheres, the high percentages of overlap shown in Table 1 indicate that the EFs shown in Fig. 3b are likely to be overestimated. In the extreme case of low concentration, when less than one particle per unit cell is present (2.4 nM), all the EFs shown in Fig. 3b will be accurate.

Comparing the overlap after 1 ms at 0.024 μM and 100 mW μm$^{-2}$ (13%) with overlap at 0.23 μM at 20 mW μm$^{-2}$ (11%), we might expect the EF for the lower power to be more accurate. However, close examination of the random draws at low concentration, but high power, shows that $\sim 50$% of the draws are without any overlap, i.e., most of the overlap occurs in a very selective region. Therefore in half of the illuminated unit cell, the EF of Fig. 3b is correct. For concentrations up to 0.024 μM and a trapping time of 1 ms, we estimate a lower bound for the EF of $10^4$.

**Table 1:** The percentage of overlapping particles after optical trapping.

Input power mW μm^−2^	Concentration μM	% overlap after 1 ms	% overlap after 10 ms
20	2.3	21	55
	0.23	11	51
	0.024	4.7	48
50	2.3	44	80
	0.23	33	74
	0.024	9.5	37
100	2.3	49	88
	0.23	38	86
	0.024	13	54

### Verifying absence of trapping away from dimer surface

Since optical trapping could occur at any position where the field gradient is high, we tested whether trapping occurred at a location in the liquid far from the substrate. As a first approximation, far from the substrate, we assume that the substrate acts as gold mirror. If we also assume that both the mirror and liquid are lossless, the standard solution of a standing wave above a perfect electric conductor (PEC) is obtained:

1 $$\begin{aligned} E(z)=2 \sin (k_{z,{\mathrm{f}}} z)E_{\mathrm{in}}, \end{aligned}$$

where $k_{z,{\mathrm{f}}}$ is the wavenumber in the fluid, and $E_{\mathrm{in}}$ is the intensity of the incident plane wave electric field. From this, it can be seen that the maximum field enhancement, which can be obtained away from the substrate, is 2 and therefore much lower than the maximum field enhancement near the nanopillar dimers. Any trapping of particles, away from the substrate, would thus greatly reduce the EF. The maximum field-gradient magnitude $\nabla |E|^2_{\mathrm{max}}$ for this solution is given by:

2 $$\begin{aligned} \nabla |E|^2_{\mathrm{max}}=4k_{z,{\mathrm{f}}}E_{\mathrm{in}}^2. \end{aligned}$$

Using Eq. 2, the optical force on a particle far from the substrate under 100 mW μm^−2^ illumination is 11.7 fN. The random thermal force vector ($F_T$) on the particle is described by a stationary stochastic process with a mean and auto-correlation function:

3 $$\begin{aligned} E\left( F_T\left( t\right) \right)&=0, \end{aligned}$$

4 $$\begin{aligned} E\left( F_T\left( t\right) F_T\left( t'\right) \right)&=2{\mathcal {D}}\delta \left( t-t'\right) . \end{aligned}$$

Here ${\mathcal {D}}=6\pi \eta r_{\mathrm{p}}k_{\mathrm{B}} T$, where $\eta$ is viscosity, $r_{\mathrm{p}}$ is the particle radius, $k_{\mathrm{B}}$ is the Boltzmann constant, and *T* is temperature. Note that ${\mathcal {D}}$ is not identical to the diffusion coefficient of the Stokes-Einstein equation, since it is no longer related to the diffusion directly, but to the force that causes the diffusion. From these properties, it can be found that the expected magnitude of the force vector is given by:

5 $$\begin{aligned} \sqrt{E\left( F_T\left( t\right) F_T\left( t\right) \right) }=\sqrt{12\pi \eta r_{\mathrm{p}} k_{\mathrm{B}} T}. \end{aligned}$$

In the present case, for particles ($\normalsize r_{\mathrm{p}}= 9{\mathrm{ nm}}$) suspended in water ($\eta = 0.890$ cP) at room temperature ($T = 298$ K), a force of 35.0 fN is obtained. Since the thermal force is approximately 3 times larger than the maximum optical force at a position far from the substrate, no trapping will occur there. Therefore, no additional correction on EF is required.

### Effect of input power on optical trapping and EF

For conventional optical trapping methods in bulk liquid, higher power generally should increase the number density of trapped particles. But is higher power better for our system? Here we discuss unexpected effects of optical power on trapping efficiency due to the shape of the surface.

Once the conditions, under which the obtained EF are valid, have been determined, a closer look can be taken at the effect of the input power on the system. As noted in the Results section, the EF for SERS in water is lower than in air due to reduced dielectric contrast. However, as shown in Fig. 5, after the particles have been under the influence of the field for 10 ms, the EF shows 1–2 orders of magnitude increase compared to one without trapping in Fig. 3b, with an additional dependence on the input power. After only 1 ms, the EF in water is similar to the surface enhancement in air, showing a minimum EF of $\sim 10^4$ at 1800 cm$^{-1}$. It appears that the gain in EF produced by the trapping of particles has less effect at higher Raman shifts. This is likely due to changes in the field distribution, with the SERS emission hotspots shifted in spatial position with respect to the hotspots of the incident 785 nm wavelength, as demonstrated in Fig. 2.

When the trapping simulations are run for 10 ms the EF is almost always larger than the surface EF in air, for all incident excitation powers. Remarkably, however, we find that the EF at 20 mW μm^−2^ is higher than at 100 mW μm^−2^ (Fig. 5). This can be traced back to the field gradient on the outer edge of the dimers. At the lower power, the gradient forces acting on particles in the high-field regions at the outer edges of the dimer, are not strong enough to overcome the thermal fluctuations. This allows particles to escape from these local field maxima, allowing the particles to sample the significantly higher-field hotspot between the dimers. For 50 mW μm^−2^ and above, the majority of particles become trapped in these local potential minima, and therefore do not benefit from the largest enhancement in the dimer gap. The optical forces for the different input powers can be found in the Supporting Information.

### Spectral shift

As shown in Fig. 6b, a shift in the AFE surface lattice resonance (SLR) peak of +0.7 nm was observed when placing a small ellipsoid between the pillars of the dimer, and this shift resulted in a decrease in the EF of about 1 order of magnitude. Such a reduction could be mitigated by modulating the laser source to increase its bandwidth, but this is undesirable for the SERS application, since it would reduce the spectral resolution. Another option would be to use a tunable laser to keep the dimers on resonance. A simpler alternative would be tilting the illumination with respect to the substrate during the measurement. It is a well known property of SLRs that they blue shift with an increase in angle^22^, and this could be used to compensate the red shift due to particle concentration.

### Application to crystal nucleation

As noted in the Introduction, a novel application of the proposed dielectric dimer array would be to induce crystal nucleation by field-enhanced trapping^10–12^. In the two-step nucleation model for crystal nucleation, dense, nanometer-scale, liquid-like droplets or clusters are considered to form spontaneously. It is believed that these species transform to become crystal nuclei^23,24^. The electric-field gradient formed by focusing a laser can act to attract and concentrate these clusters, even if they are too small to trap^25^.

Niinomi et al. demonstrated crystal nucleation by focusing a continuous-wave (CW) laser (1064 nm, 20 mW) onto a 2D Au-nanolattice immersed in an aqueous solution of acetaminophen (the popular analgesic)^11^. Crystals formed after $\sim {100}\space{\mathrm {ms}}$ in an annular ring approximately $19$ μm outside the $2$ μm-diameter focal spot. The ring is formed because of a balance between the localized electric-field gradient force (attractive) and thermophoretic force (repulsive) due to local heating. This high degree of control over crystallization not only offers fundamental insights into nucleation mechanisms, it shows great potential for discovery of new crystal polymorphs, e.g., as desired by the pharmaceutical industry^26–28^.

### Biological applications

Due to their small size and relatively low concentration in the body, viruses can be difficult to detect spectroscopically. Such species can also be highly sensitive, even to moderate temperatures ($> 37\space^\circ$C). By concentrating the virus analyte particles in the high field regions of the nanostructure, the boosted signal could facilitate identification by increasing the signal-to-noise ratio of the Raman spectrum. For example, Polio is a small enterovirus, approximately 25–30 nm in diameter (refractive index, *n* = 1.61 ± 0.06) which could be localized for enhanced detection by Raman spectroscopy^18^. The use of plasmon-based SERS sensors, coupled with data analysis strategies employing artificial intelligence methods, have been emphasized in particular for SARS-Cov-2, the virus responsible for the Covid-19 pandemic^14^.

## Conclusions

In summary, it has been shown that a dielectric dimer array, with free analyte particles in aqueous solution, can achieve higher SERS EFs than an equivalent array in air, with fixed analyte particles. The higher EFs are due to optical trapping of particles, which localize around the electric-field hotspots. The results demonstrate a maximum obtainable EF of $1.5\times 10^5$ after 10 ms of optical trapping, approximately 275% higher than can be obtained with a dry substrate. For hard particles, this high EF is only obtained at low concentrations ($\sim 2.4$ nM). At higher concentrations, the particles compete and the electric-field hotspots become saturated: excess particles tend to localize in positions of lower electric field.

For concentrations up to 0.024 μM and a trapping time of 1 ms, a lower bound for the worst case maximum enhancement of $10^4$ was estimated. This is approximately 25% of the best obtainable EF for a dried substrate. It was shown that increasing the illumination power does not necessarily lead to higher EFs. For intensities higher than 20 mW μm^−2^ particles became trapped in local potential minima, where the EF is lower than the electric-field hotspot between the dimer pillars. For SERS applications it is therefore recommended to use immersed dimers for low analyte concentrations at low incident laser powers.

A promising advantage of the immersed dielectric dimer array configuration detailed here is the relative reduction in heating due to absorption compared to noble metal substrates. For this reason, a novel application of the immersed dimer arrays could be laser-induced crystal nucleation, where the electric-field hotspots act to increase the local concentration of solute molecules and clusters particles. Moreover, the array configuration shows tremendous promise for localizing and enhancing SERS signals from biological macromolecules, such as proteins and viruses.

## Methods

### Simulation of electric-field enhancement

The electric field distribution was simulated using COMSOL Multiphysics Version 5.4. An optical plane-wave input was applied to a port at the top of the unit cell, with an empty port on the bottom (i.e., along the *z* direction, see Fig. 1). Both ports are periodic, with periodic boundary conditions also applied to the faces of the unit cell in the *x* and *y* directions. The scattered field was simulated for input wavelengths 750 to 950 nm in increments of 1 nm, with the power normalized to produce a background field of 1 V m^−1^. The scattered field was used to compute the enhancement factor. An optical plane-wave input of wavelength 785 nm and power of 5 mW μm^−2^ was used to simulate the scattered field when calculating the dielectrophoretic force on a particle in proximity to the metasurface. A multiplier was included to the force to model an optical field with power inputs of 20, 50 and 100 mW μm^−2^, respectively.

### Simulation of optical trapping

A particle-tracing algorithm was built in MATLAB R2019a, using velocity Verlet-type differential equations, modified to keep the energy of the system from diverging due to the timestep-dependence of Brownian motion, as described by Grønbech-Jensen et al.^29^.

6 $$\begin{aligned} r^{\{n+1\}}=\space & {} r^{\{n\}} + b\,{\mathrm{d}}t\,v_{\mathrm{p}}^{\{n\}}+ \frac{b\,{\mathrm{d}}t^2}{2 m_{\mathrm{p}}} F^{\{n\}} + \frac{b\,{\mathrm{d}}t}{2 m_{\mathrm{p}}} \beta ^{\{n\}} \end{aligned}$$

7 $$\begin{aligned} v_{\mathrm{p}}^{\{n+1\}}= \space& {} v_{\mathrm{p}}^{\{n\}} + \frac{{\mathrm{d}}t}{2 m_{\mathrm{p}}} \left( F^{\{n\}} + F^{\{n+1\}}\right) - \frac{{\alpha}}{m_{\mathrm{p}}} \left( r^{\{n+1\}} -r^{\{n\}}\right) + \frac{\beta ^{\{n+1\}} }{m_{\mathrm{p}}} \end{aligned}$$

The terms are explained in Table 2.

**Table 2:** Definition of all variables used in the optical trapping algorithm.

**Variable**	**Definition**
$r^{\{n+1\}}$	Position at timestep $n+1$
$r^{\{n\}}$	Position at timestep *n*
$b = \left( 1 + \alpha \,{\mathrm{d}}t/2 m_{\mathrm{p}}\right) ^{-1}$	Correction term
$\alpha = m_{\mathrm{p}}/\tau _{\mathrm{p}}$	Friction coefficient
$\tau _{\mathrm{p}}$	Relaxation time
$v_{\mathrm{p}}^{\{n\}}$	Particle velocity at timestep *n*
$F^{\{n\}}$	Total external force (excluding drag and random thermal fluctuations) at timestep *n*
$\beta = r \sqrt{2 \alpha k_B T / {\mathrm{d}}t}$	Thermal white noise
*T*	Temperature (298 K)
$k_{\mathrm{B}}$	Boltzmann constant
${\mathrm{d}}t$	Timestep

A timestep size of $10^{-9}$ s was chosen because, for a nanometer scale particle in free space, the average velocity is of the order 1 m s$^{-1}$, so this gives a position shift of $\sim$ 1 nm per timestep. The total external force can be expressed as $F^{\{n\}} = F_{\mathrm{e}}^{\{n\}}+F_{\mathrm{g}}$ where $F_{\mathrm{e}}$ is the dielectrophoretic force given in Eq. 8 and $F_{\mathrm{g}}$ is the force due to gravity given in Eq. 9.

8 $$\begin{aligned} F_{\mathrm{e}} = 2 \pi r_{\mathrm{p}}^3 \varepsilon _0 {\text {Re}}(\varepsilon _{\mathrm{f}}) {\text {Re}} \bigg (\frac{\varepsilon _{\mathrm{p}} - \varepsilon _{\mathrm{f}}}{\varepsilon _{\mathrm{p}} + 2 \varepsilon _{\mathrm{f}}}\bigg ) \nabla |E|^2 \end{aligned}$$

The dielectrophoretic force is dependent on the gradient of the square of the field magnitude, $\nabla |E|^2$, the particle radius, $r_{\mathrm{p}}$, and dielectric constant of the particle, $\varepsilon _{\mathrm{p}}$, and surrounding fluid, $\varepsilon _{\mathrm{f}}$.

9 $$\begin{aligned} F_{\mathrm{g}} = m_{\mathrm{p}} g\bigg (\frac{\rho _{\mathrm{p}} - \rho _{\mathrm{f}}}{\rho _{\mathrm{p}}}\bigg ) \end{aligned}$$

where $m_{\mathrm{p}}$ is the particle mass, *g* is the gravitational acceleration 9.81 ms$^{-2}$, $\rho _{\mathrm{p}}$ is the density of the particle and $\rho _{\mathrm{f}}$ is the density of the surrounding fluid.

The scattered field generated in COMSOL was imported to MATLAB, and periodic boundary conditions were implemented in the *x* and *y* directions to model the infinite substrate, so a particle that leaves the unit cell would re-enter from the opposite side. Elastic collision conditions were implemented on the substrate and dimer surfaces.

### Computation of enhancement factor

The study by Černigoj et al. defined EF as Eq. 10^8^. This is the total enhancement factor, integrated over the substrate surface.

10 $$\begin{aligned} \text {EF}(\omega _0,\omega _{\text {R}}) = \frac{1}{A}\mathop {\int }\limits _S \bigg | \frac{E_{\text {loc}}(\omega _{0},{\varvec{r}})}{E_0}\bigg |^2 \cdot \bigg |\frac{E_{\text {loc}}(\omega _{\text {R}},{\varvec{r}})}{E_0}\bigg |^2 \,dS \end{aligned}$$

$\text {EF}(\omega _0,\omega _{\text {R}})$ is the enhancement factor averaged over the whole substrate surface, *A* is the surface area of the substrate, $E_{\text {loc}}(\omega _{0},{\varvec{r}})$ is the electric field magnitude at incident frequency $\omega _0$, and point ${\varvec{r}}$ on the surface, $E_{\text {loc}}(\omega _{\text {R}},{\varvec{r}})$ is the electric field magnitude at emission frequency $\omega _{\text {R}}$ and point ${\varvec{r}}$ on the surface, and $E_0$ is the background field magnitude, before enhancement.

In fluid, Eq. 10 must be modified to integrate over the fluid volume, *V*, surrounding the dimers in the unit cell, as Eq. 11.

11 $$\begin{aligned} \text {EF}(\omega _0,\omega _{\text {R}}) = \frac{1}{V}\mathop {\int }\limits _V \bigg |\frac{E_{\text {loc}}(\omega _{0},{\varvec{r}})}{E_0}\bigg |^2 \cdot \bigg |\frac{E_{\text {loc}}(\omega _{\text {R}},{\varvec{r}})}{E_0}\bigg |^2 \,dV \end{aligned}$$

To obtain the EF from the metasurface while taking into account trapping, a regular array of 9 × 9 × 12 particles (972 particles) was released within the unit cell, and the enhancement factor computed at initial positions, after 1 ms and 10 ms. Instead of integrating over the whole volume, the summation over the local field enhancement for each particle was used, as in Eq. 12.

12 $$\begin{aligned} \text {EF}(\omega _0,\omega _{\text {R}}) = \frac{1}{N}\mathop {\sum }\limits _N \bigg |\frac{E_{\text {loc}}(\omega _{0},{\varvec{r}}(t))}{E_0}\bigg |^2 \cdot \bigg |\frac{E_{\text {loc}}(\omega _{\text {R}},{\varvec{r}}(t))}{E_0}\bigg |^2 \end{aligned}$$

where *N* is the number of particles, and ${\varvec{r}}(t)$ in this case is the position of each particle within the volume at time *t* after release from uniform distribution.

### Optimization of structure

The metasurface design of Černigoj et al. was used as a starting point: however, this surface was optimized to produce maximum field enhancement when the surrounding medium is air. For the equivalent optimization in water, as required for the mobility of particles influenced by the electric field gradient, the dimensions must be adjusted due to the different refractive index of water. The period of the dimer unit cells was divided by the refractive index of water at 785 nm ($n = 1.33$), with minor adjustments made to produce maximum enhancement. This gives a unit cell with side length 556 nm. Keeping the 20 nm gap between the nanocylinders, their dimensions (height and diameter) were adjusted, using a factor of the refractive index ratio between silicon and air, and silicon and water, both at 785 nm (a factor of 1.13). Minor further numerical adjustments were made to the input parameters to achieve maximum enhancement, giving optimized dimers with height 333 nm, and diameter 139 nm.

## Supplementary Information

Supplementary Information.
