# Supplementary material for: Using the near field optical trapping effect of a dielectric metasurface to improve SERS enhancement for virus detection
Source: Sci Rep. 2021 Mar 25;11:6873. doi: 10.1038/s41598-021-85965-1 (PMC7994300; doi:10.1038/s41598-021-85965-1)
Supplement: Supplementary file 1 — Supplementary Information. [file 41598_2021_85965_MOESM1_ESM.pdf]

Supplementary information for Using the Near Field Optical Trapping Effect of a Dielectic Metasurface to Improve SERS Enhancement for Virus Detection

Cameron F. Kenworthy, L. Pjotr Stoevelaar, Andrew Alexander, Giampiero Gerini

ABSTRACT

Particle Distribution

The distribution of particle distance from the hotspot after 1 ms and 10ms are shown in fig.S1. As can be seen from the plots, some trapping has occurred after 1 ms. The peak at the bin 0-50 nm represents particles in the gap between the dimers. The peak at the bin 150-200 nm represents particles stuck against the sides of the cylinders. The bins with distance greater than 200 nm represent untrapped particles. If we use this division in trapped and untrapped particles, it is found that after 1ms 39.9% of the particles are trapped and after 10ms 79.8% of the particles are trapped.

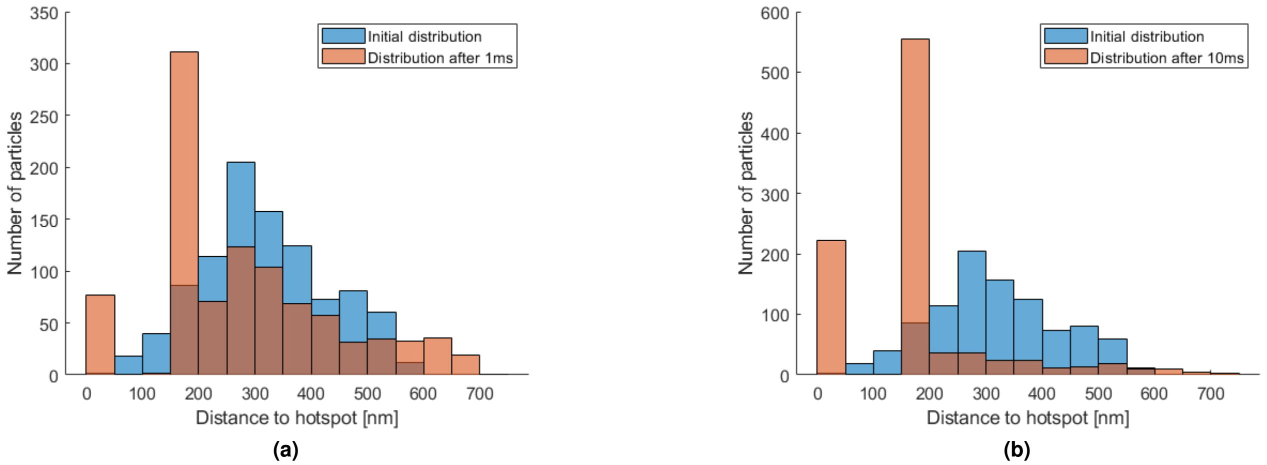

Figure S1. The distribution of particle distance from the hotspot. (a) 1 ms of illumination. (b) 10 ms of illumination.

Absorption

As can be seen in Fig. S2, the absorptance of the dimers in liquid reaches a maximum of 34% at the laser excitation wavelength of 785 nm, and a second peak of 13% occurs at 830 nm wavelength, at the dimer resonance. The high absorption at the laser excitation wavelength, could lead to an increase in temperature of the water: thus, increasing the thermal forces acting on the particles and reducing the trapping efficiency of the structure. However, as is shown in Fig.S3, at both the excitation wavelength and the dimer resonance, the majority of the absorption occurs in the gold substrate. Since gold has a high thermal conductivity (310 W m^{-1} K^{-1}) compared to water (0.6 W m^{-1} K^{-1}) and silicon and silicon-dioxide (1.3 W m^{-1} K^{-1}), proper cooling via the back of the gold substrate can mitigate the increase of temperature of the water.

Optical forces

As was shown in the main text, the highest enhancement factor was not necessarily obtained for the highest laser power. This can be explained by reference to Fig. S4. These figures show the field intensity in the cross section corresponding to the

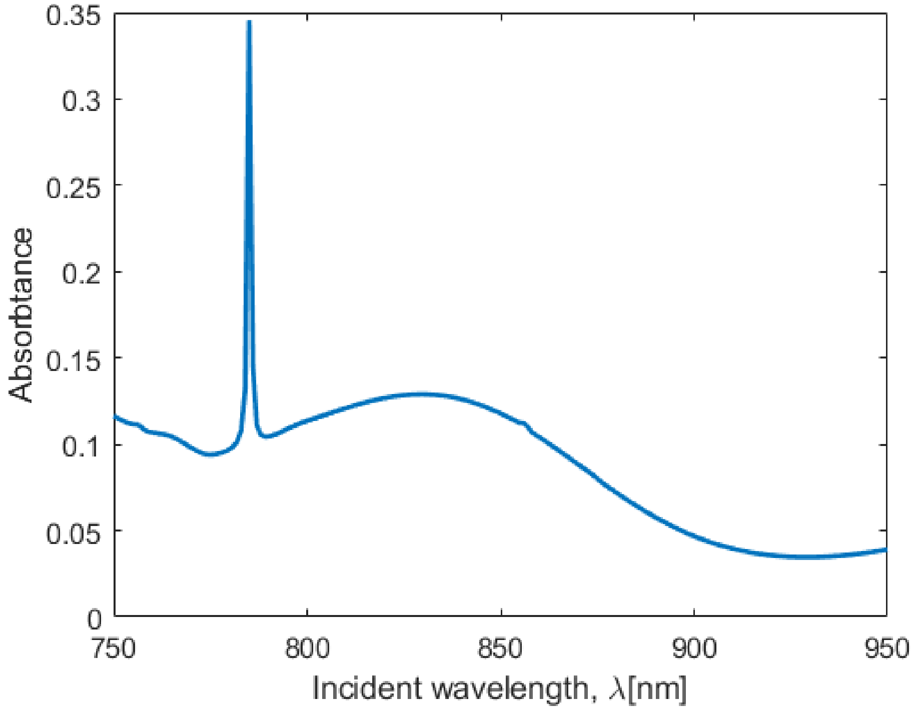

Figure S2. Absorptance of dimer-array immersed in water, as function of wavelength

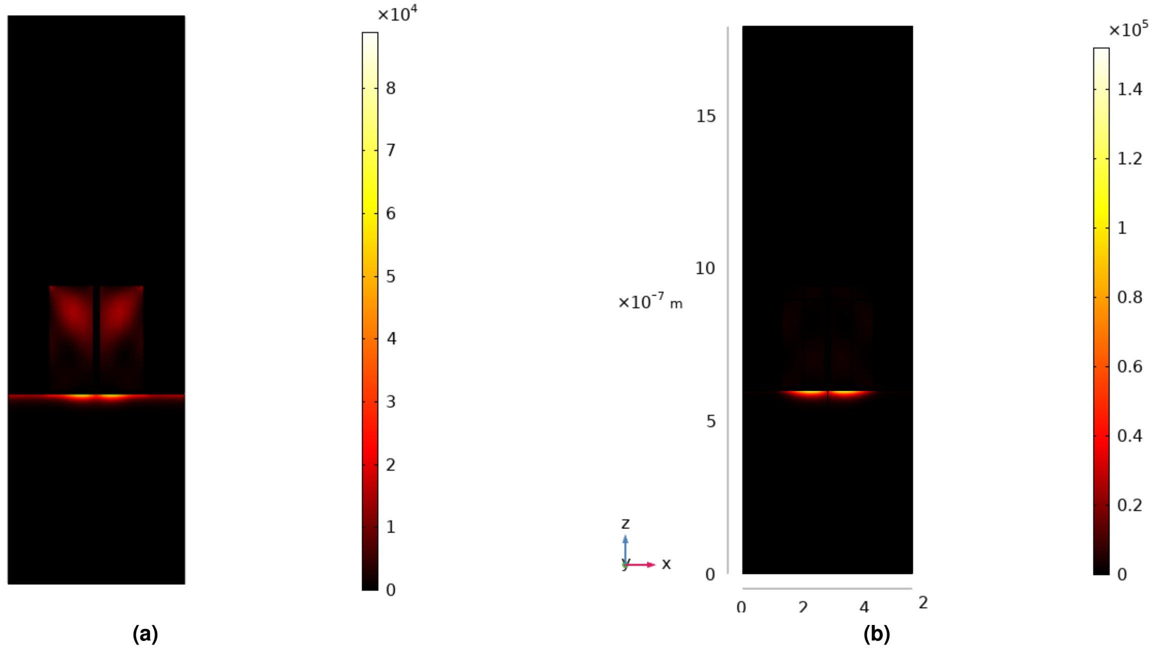

Figure S3. (a) Absorption density W m^{-3} inside the dimer unit-cell for an incident wavelength of 785 nm. (b) Absorption density inside the dimer unit-cell W m^{-3} for an incident wavelength of 850 nm.[1]

polarization plane of the incident electromagnetic field, for different values of the excitation power. It can be observed that the highest field enhancement is always concentrated in the higher part of the dimers. Nevertheless, the optical trapping volume, where the optical forces (attractive) are stronger than the thermophoretic forces (repulsive), include a larger portion of the

dimers. The white contour lines define exactly this region. As it can be observed, such region tends to extend also to the lower part of the dimers, when the power is increased. Since in this lower lower part, the optical force is directed towards the bottom of the structure (white arrow), the particles tend to remain trapped there, but they experience a much less strong field enhancement. Consequently, we can conclude that the overall number of particles trapped in the area of stronger field enhancement decreases with larger values of the excitation power, therefore explaining the observed behavior of the EF.

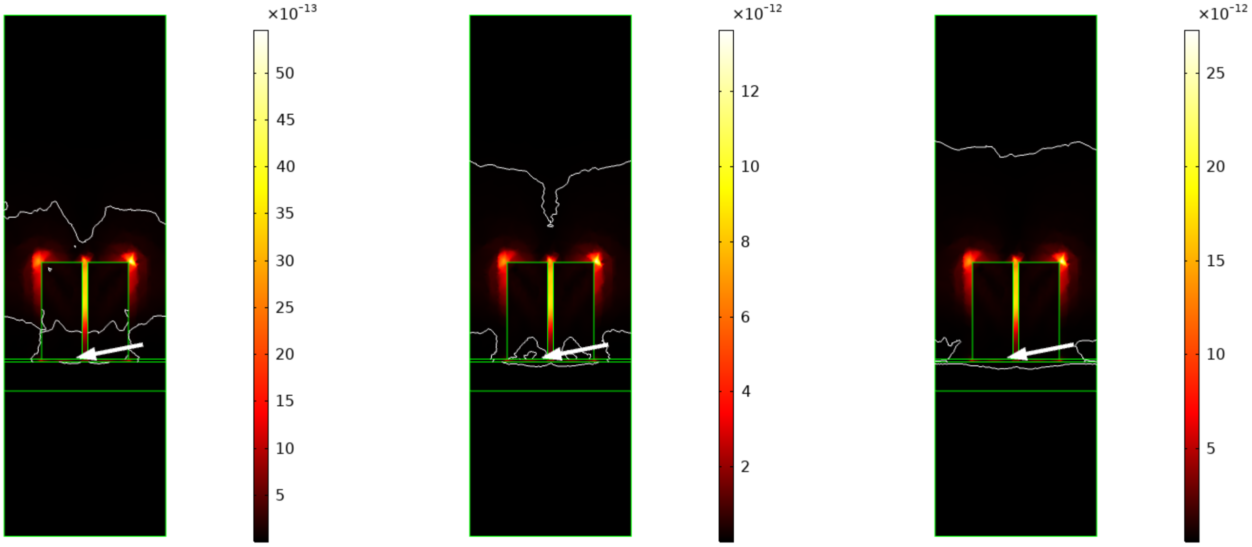

(a) (b) (c)

Figure S4. Optical force for different illumination intensities I. The white line indicates the boundary between the zone where the optical force is greater than the thermal one. The arrow indicates the direction of the optical force: (a) $I = 20$ mW μm^{-2}; (b) $I = 50$ mW μm^{-2}; (c) $I = 100$ mW μm^{-2}.[1]

References

1. COMSOL. Multiphysics (5.4a) (2020). "https://www.comsol.com/".
